# Supplementary figures and images for: Genomic fingerprints of the world’s soil ecosystems
Source: mSystems. 2024 May 9;9(6):e01112-23. doi: 10.1128/msystems.01112-23 (PMC11237643; doi:10.1128/msystems.01112-23)

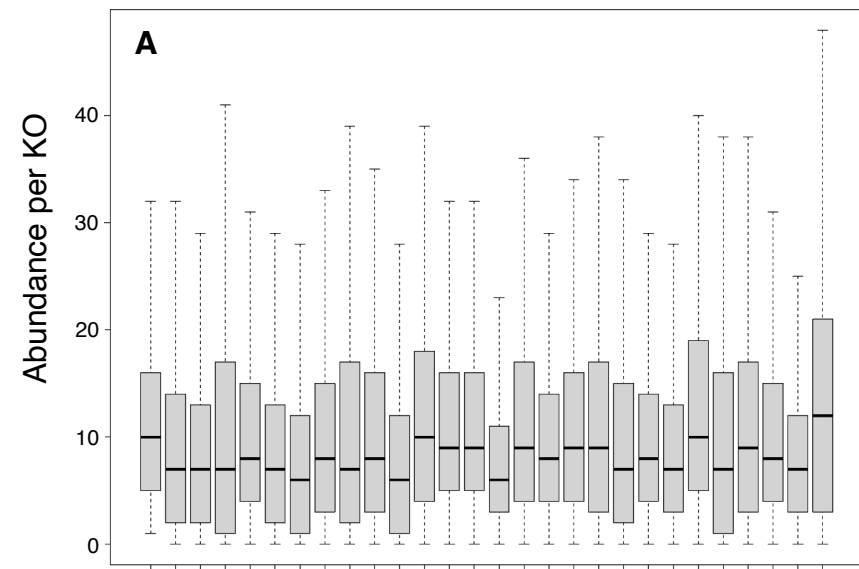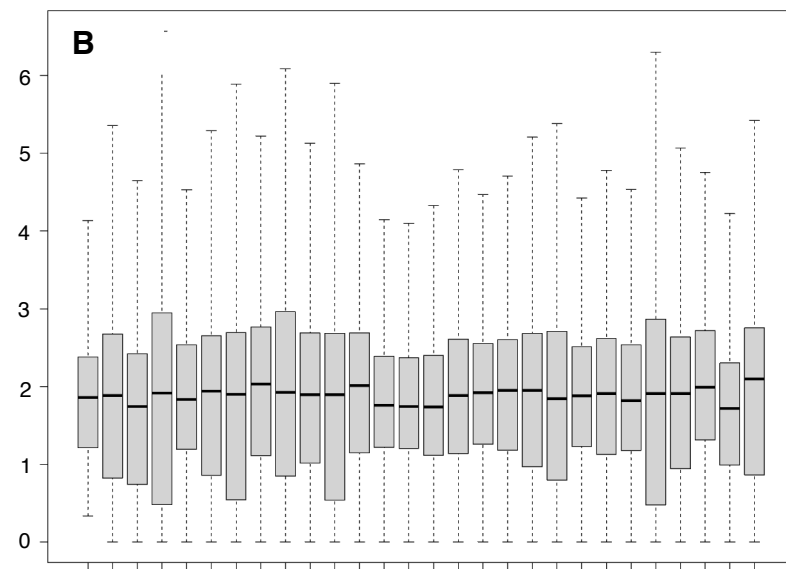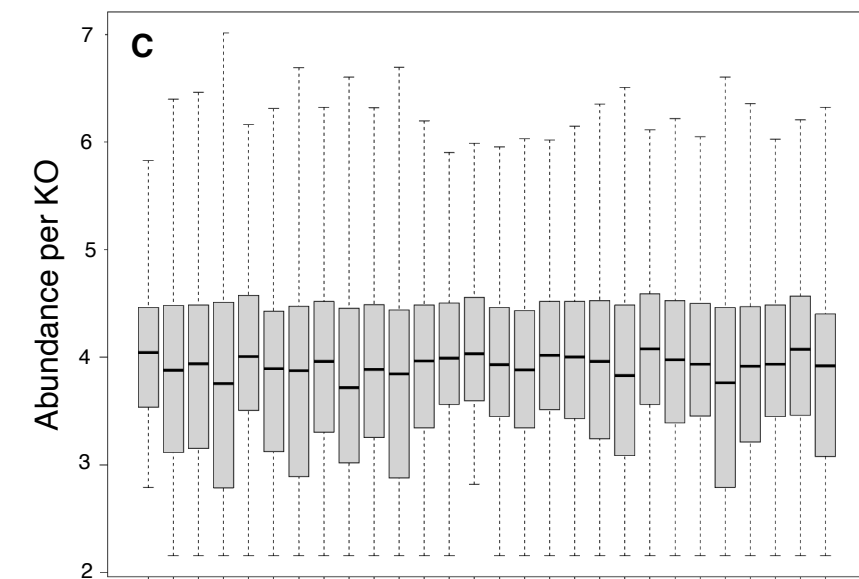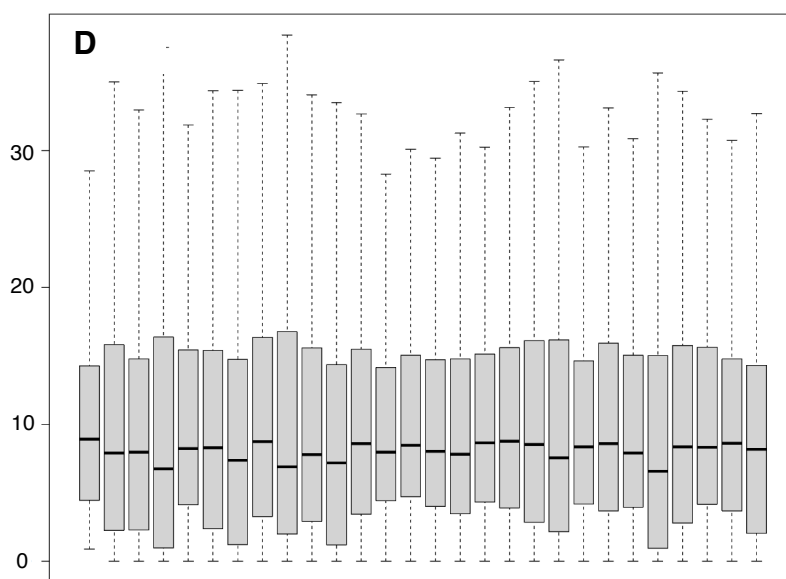

Supplement: Figure S1 — Abundances across different normalization procedures. [file msystems.01112-23-s0003.pdf]

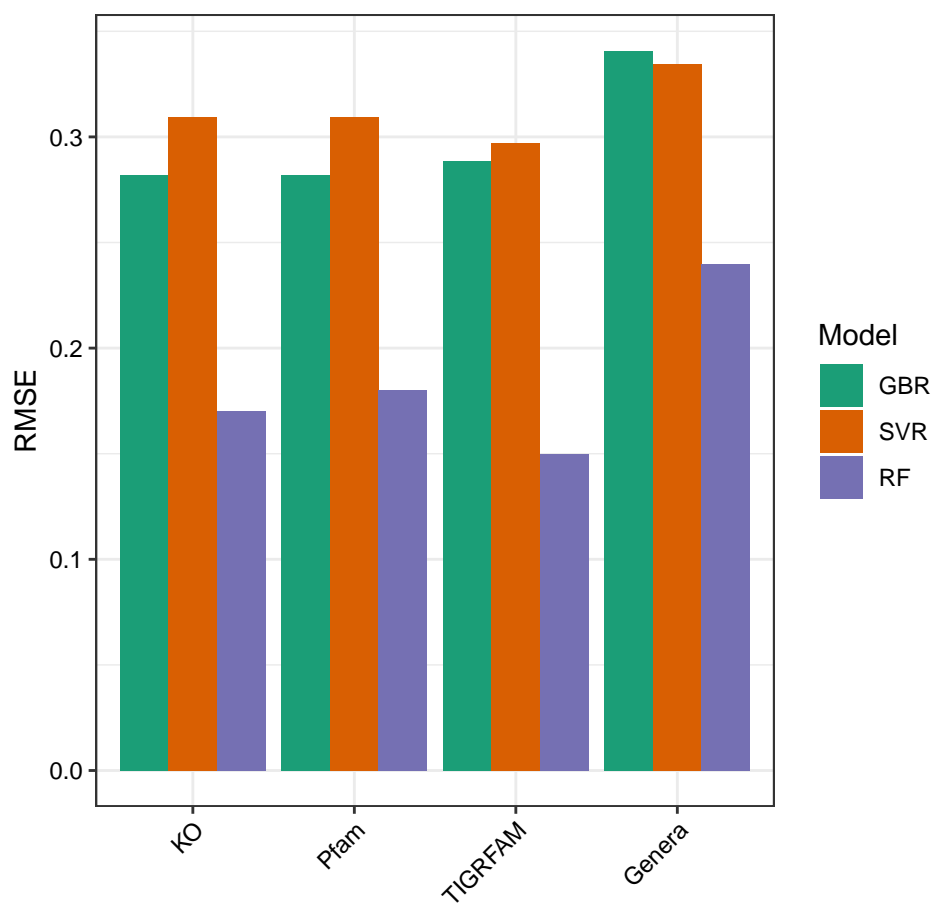

Supplement: Figure S2 — Comparison of machine learning algorithms. [file msystems.01112-23-s0004.pdf]
